# A rare case of septic pulmonary embolism in co-existence with infective endocarditis and COVID-19

**DOI:** 10.2217/fvl-2022-0029

**Published:** 2022-07-19

**Authors:** Yusuf Emre Ozdemir, Adile Sevde Demir, Meryem Sahin Ozdemir, Busra Mavi, Can Ozen, Hayat Kumbasar Karaosmanoglu

**Affiliations:** ^1^Department of Infectious Diseases & Clinical Microbiology, Bakirkoy Dr Sadi Konuk Training Research Hospital, Istanbul, 34140, Turkey; ^2^Department of Infectious Diseases & Clinical Microbiology, Istanbul University – Cerrahpasa, Cerrahpasa Faculty of Medicine, Istanbul, 34093, Turkey,; ^3^Department of Cardiology, Bakirkoy Dr Sadi Konuk Training Research Hospital, Istanbul, 34140, Turkey; ^4^Department of Cardiovascular Surgery, Bakirkoy Dr Sadi Konuk Training Research Hospital, Istanbul, 34140, Turkey

**Keywords:** anticoagulant therapy, COVID-19, infective endocarditis, methicillin sensitive *Staphylococcus aureus*, septic pulmonary embolism

## Abstract

Infective endocarditis (IE) symptoms including fever, fatigue, dyspnea and myalgia are similar in COVID-19 findings. Therefore, the diagnosis of IE may be missed in patients with COVID-19. Co-existence with IE in COVID-19 is rarely reported. However, to our knowledge, only one case of septic pulmonary embolism in COVID-19 and IE was reported. Here, we describe a case of septic embolism due to tricuspid endocarditis caused by intravenous drug use in patients with COVID-19. In this fatal case, the use of prophylactic anticoagulants due to COVID-19 probably caused the tendency to hemorrhagic cerebrovascular complications. Our report emphasizes the complexity of anticoagulant prophylaxis in patients with COVID-19 which may cause hypercoagulopathy in co-existence with IE.

COVID-19 caused by SARS-COV-2, continues to affect the world and result in many deaths. Although COVID-19 mainly involves the respiratory system, a wide variety of clinical findings associated with other system involvement have been reported [1]. Many cardiovascular complications such as arrhythmia, coronary artery syndrome, myocarditis, pulmonary embolism, heart failure, pericarditis and infective endocarditis (IE) have been reported [2]. IE may present with fever, fatigue, shortness of breath and myalgia or may progress with a silent clinical picture. Diagnosis of IE may be missed in patients with COVID-19 [3]. Therefore, patients with risk factors for IE should also be evaluated according to Duke’s criteria [4].

In this report, we present a rare case of a septic pulmonary embolism due to tricuspid valve endocarditis in a SARS-CoV-2 real-time PCR (RT-PCR)-positive refugee who was an intravenous drug user hospitalized with fever, chills and myalgia.

## Case report

**Figure 1.**
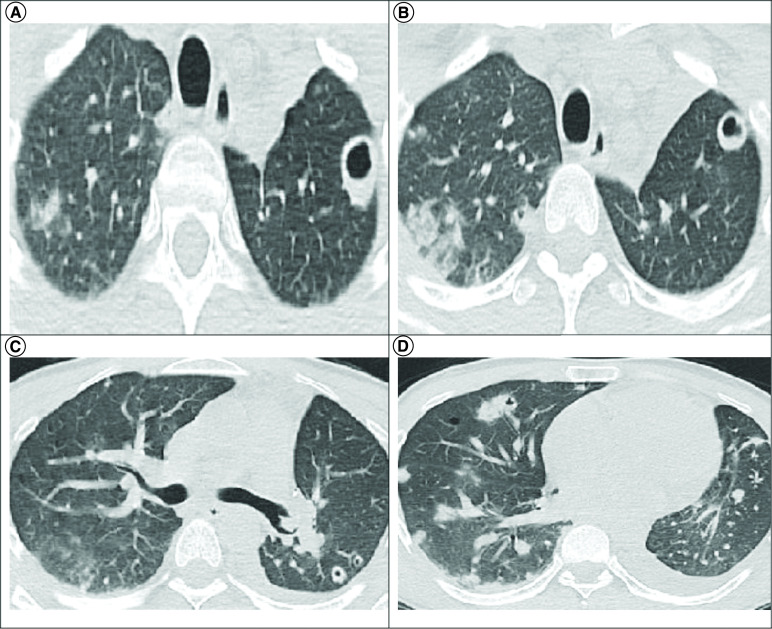
Chest computed tomography images of the patient.

A 45-year-old male with no known medical history applied to the emergency department with fever, chills, widespread myalgia and arthralgia for 8 days. On admission, the patient was alert, his blood pressure was 114/60 mmHg, heart rate 86 bpm, body temperature 38.1°C, respiratory rate 20 breaths per min and peripheral capillary oxygen saturation at 95%. Initial laboratory parameters; white blood cells (WBC) 22.3 × 10^3^/ul (normal: 3.7–10.1 × 10^3^/ul), lymphocyte count 0.59 × 10^3^/ul (normal: 1.09–2.99 × 10^3^/ul), hemoglobin 10 g/dl (normal: 12.9–15.9 g/dl), platelets 111 × 10^3^/ul C-reactive protein (CRP) at 292 mg/l (normal: 0–5 mg/l), procalcitonin 1.21 ng/ml (normal: <0.5 ng/ml) (normal: 153–366 × 10^3^/ul) and d-dimer 2.36 μg fibrinogen equivalent units/ml (normal: 0–0.5 μg fibrinogen equivalent units/ml). Two sets of blood culture was obtained from the patient, and chest computed tomography (CT) was performed. Chest CT showed multiple cavitary thick-walled lesions with a largest diameter of 16 mm in the upper lobes of bilateral lungs, scattered nodular densities and peripheral predominant ground-glass opacities (Figure 1A–D).

He was admitted to the pandemic hospital because the nasopharyngeal swab sample for the SARS-CoV-2 RT-PCR test was positive. He was examined for septic embolism, tuberculosis and Wegener’s diseases. Favipiravir and low molecular weight heparin were started according to the COVID-19 Guideline of the Republic of Turkey’s Ministry of Health [5]. Acid-fast bacteria were not detected in two consecutive sputum samples. The vasculitis markers (antinuclear antibody and anti-neutrophil cytoplasmic antibody) were negative.

**Figure 2.**
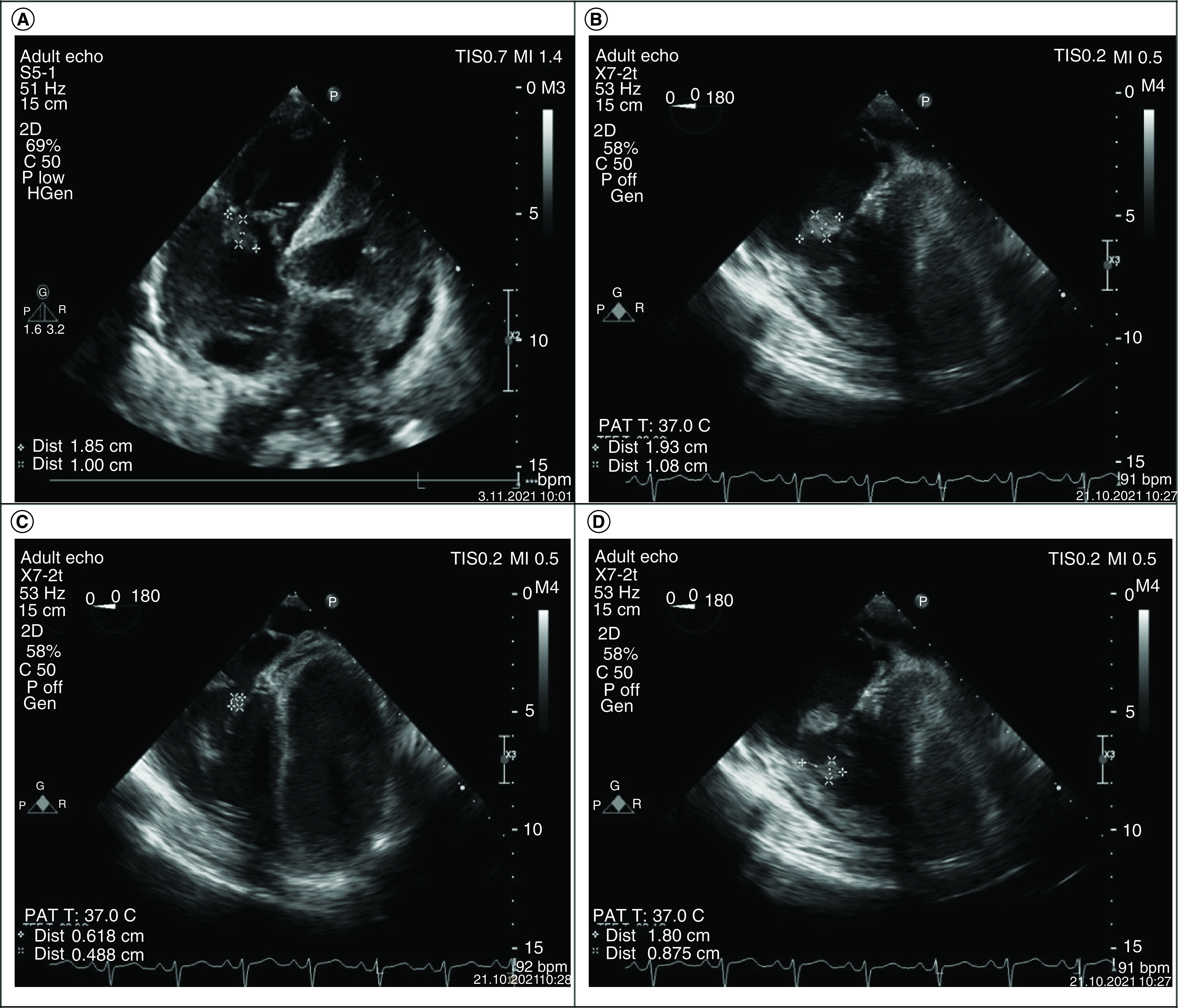
Transthoracic and transesophageal echocardiography images of the patient.

On the third day following admission, transthoracic echocardiography revealed 17 × 21^-mm^ vegetation on the tricuspid valve and mild tricuspid insufficiency (Figure 2A). Abdominal CT and cranial MRI were planned to investigate other possible embolic foci in the patient, since the vegetation was detected by transthoracic echocardiography. Hepatomegaly (220 mm) and splenomegaly (145 mm) were detected on abdominal CT. No pathology was detected in cranial MRI. Methicillin-sensitive *Staphylococcus aureus* (MSSA) was isolated from all blood-culture samples (four out of four samples); therefore, i.v. cefazolin 3 × 2 gr was started. MSSA was detected in the control blood culture on the 3rd day of the treatment. On the 6th day of the IE treatment, the control blood culture yielded a negative result. His acute phase reactants regressed (CRP: 101 mg/l, procalcitonin 0.75 ng/ml, WBC: 18.5 × 10^3^/ul). Control transesophageal echocardiography revealed 1.9 × 1^-cm^ vegetation on the tricuspid valve septal and anterior leaflet, 1 × 0.8^-cm^ vegetation on the chordae and 0.4 × 0.6^-cm^ vegetation on the septal leaflet and as well as patent foramen ovale and severe tricuspid insufficiency (Figure 2B–D).

On the 10th day of the IE treatment, the patient was evaluated by the cardiovascular surgery council and an operational decision was made. On the 12th day of IE treatment, his vital parameters were stable, with a CRP of 43 mg/l, procalcitonin of 0.24 ng/ml and WBC of 13.3 × 10^3^/ul. Control SARS-CoV-2 RT-PCR test was detected as negative, and he was transferred to the cardiovascular surgery clinic.

**Figure 3.**
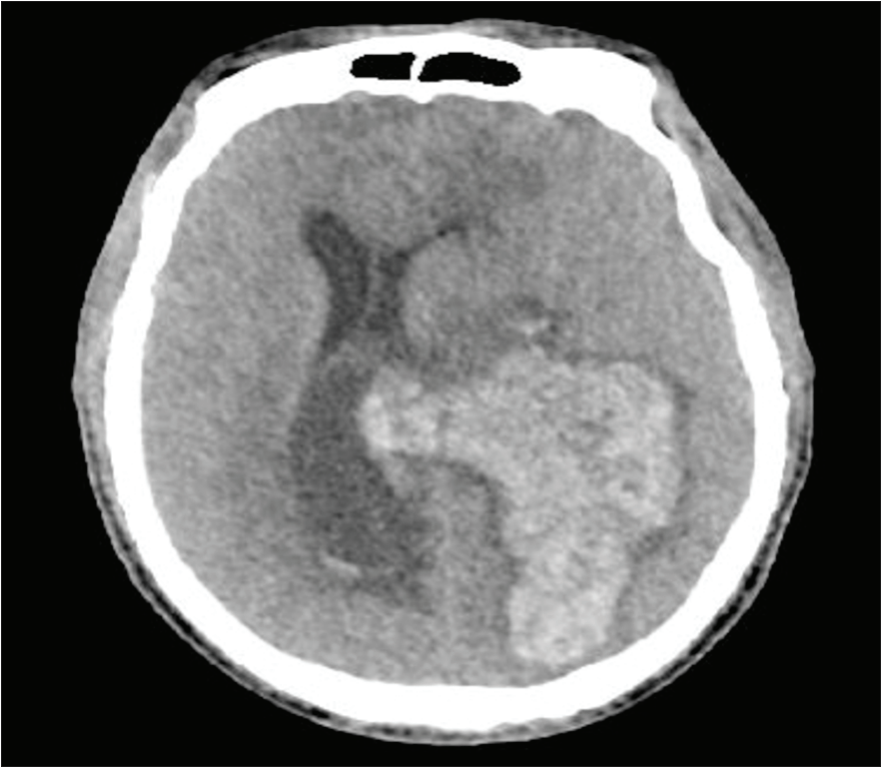
Cranial MRI of the patient after intracranial hemorrhage.

On the 25th day of IE treatment, the patient was transferred to the intensive care unit due to a sudden loss of consciousness and anisocoria. Cranial CT showed a hematoma area measuring 84 × 43 mm in its widest part and a thin linear hypodensity consistent with edema in the parietal lobe of the left cerebral hemisphere. The left lateral ventricle was compressed, and a 17-mm rightward shift was observed in the midline structures (Figure 3). The patient underwent emergency craniectomy and hematoma evacuation. He died on the second postoperative day from cardiac arrest in the intensive care unit following the operation.

## Discussion

Co-existence of COVID-19 with other diseases will be more common in daily practice due to COVID-19 pandemic [6]. Therefore, the clinical presentation of other infectious diseases may be confused, other possible diseases in patients with certain risk factors. In the case reported here, the patient presented with fever, chills and widespread myalgia and arthralgia. On the face of it, a diagnosis of COVID-19 can be considered reasonable for patients with positive SARS-CoV-2 RT-PCR who applied with compatible clinical findings during the pandemic period [7]. Although pulmonary cavitation in COVID-19 is rare, it has been reported in some cases [8,9]. However, other causes of pulmonary cavitation, such as IE, tuberculosis and Wegener’s diseases, were investigated, since the patient had certain risk factors, including low socioeconomic status and intravenous drug use. Two major criteria, including vegetation on the tricuspid valve and MSSA isolation from the blood culture (four out of four samples), as well as three minor criteria, including fever, septic embolism and intravenous drug use, were met with Duke’s criteria [4].

The treatment of IE varies widely according to the pathogen microorganisms, their resistance patterns and host factors (native valve/prosthetic valve). In cases with inefficient medical treatment, surgical procedure is applied. Monotherapy with nafcillin, oxacillin or cefazolin is recommended for native valve endocarditis due to MSSA [10]. Since nafcillin and oxacillin are not available in Turkey, cefazolin was preferred for treatment. Significant acute phase reactant response was obtained under cefazolin treatment. Surgical intervention was decided upon because there was no decrease in the vegetation size and advanced valve insufficiency developed in the control transesophageal echocardiography. However, elective surgery is recommended for right-valve endocarditis under the current guidelines [10–13]. In fact, it is recommended to avoid surgery if possible in intravenous drug users. In our case, the patient, who was an intravenous drug user, was hemodynamically stable. In addition, the patient’s bacteremia on the 6th day of the treatment ceased, and acute-phase reactants decreased. Therefore, the cardiovascular surgery council decided to delay the surgical operation as much as possible.

Thromboembolic complication is a serious problem in the management of IE. The available evidence for the benefits or safety of anticoagulant therapy in patients with IE is inadequate. There have been no randomized studies of anticoagulant therapy in this group [14]. It has been reported that anticoagulant therapy increases mortality by causing hemorrhagic complications [15]. On the contrary, there are studies reporting that this therapy reduces cerebrovascular events [16,17]. Due to these uncertain data, the use of anticoagulants is recommended on a patient basis by evaluating the risks and benefits of treatment [10]. In addition, it is recommended that anticoagulant therapy be continued in patients with previous indications for different reasons [10,14,18]. However, the prophylactic anticoagulant treatment recommendation for COVID-19 has also changed continuously throughout the pandemic. There is no clear recommendation for the use of anticoagulants when COVID-19 and IE coexist. Therefore, anticoagulant therapy was continued in the patient. Perhaps it would have been more appropriate to discontinue anticoagulant treatment after SARS-CoV-2 RT-PCR negativity was achieved. For this reason, detailed studies should be conducted on how to manage anticoagulant prophylaxis for COVID-19 in cases of co-existence with IE, which may be encountered more frequently during the pandemic.

## Conclusion

In conclusion, IE and its complications should be considered in COVID-19 patients with certain risk factors. The use of anticoagulants in the management of patients with COVID-19 and IE should be carefully evaluated on a patient basis.

**Executive summary**

- Infective endocarditis (IE) symptoms including fever, fatigue, dyspnea and myalgia are similar in COVID-19 findings.
- IE and its complications should be considered in COVID-19 patients with certain risk factors.
- Clinicians should be aware of that systemic embolisms may develop in addition to pulmonary septic embolisms in right valve IE in patients with patent foramen ovale.
- The available evidence for the benefits or safety of anticoagulant therapy in patients with IE is inadequate.
- The use of anticoagulants in the management of patients with COVID-19 and IE should be carefully evaluated on patient basis.
